# A Measure of Information Available for Inference

**DOI:** 10.3390/e20070512

**Published:** 2018-07-07

**Authors:** Takuya Isomura

**Affiliations:** Laboratory for Neural Computation and Adaptation, RIKEN Center for Brain Science, 2-1 Hirosawa, Wako, Saitama 351-0198, Japan; takuya.isomura@riken.jp; Tel.: +81-48-467-9644

**Keywords:** free-energy principle, internal model hypothesis, unconscious inference, infomax principle, independent component analysis, principal component analysis

## Abstract

The mutual information between the state of a neural network and the state of the external world represents the amount of information stored in the neural network that is associated with the external world. In contrast, the surprise of the sensory input indicates the unpredictability of the current input. In other words, this is a measure of inference ability, and an upper bound of the surprise is known as the variational free energy. According to the free-energy principle (FEP), a neural network continuously minimizes the free energy to perceive the external world. For the survival of animals, inference ability is considered to be more important than simply memorized information. In this study, the free energy is shown to represent the gap between the amount of information stored in the neural network and that available for inference. This concept involves both the FEP and the infomax principle, and will be a useful measure for quantifying the amount of information available for inference.

## 1. Introduction

Sensory perception comprises complex responses of the brain to sensory inputs. For example, the visual cortex can distinguish objects from their background [1], while the auditory cortex can recognize a certain sound in a noisy place with high sensitivity, a phenomenon known as the cocktail party effect [2,3,4,5,6,7]. The brain (i.e., a neural network) has acquired these perceptual abilities without supervision, which is referred to as unsupervised learning [8,9,10]. Unsupervised learning, or implicit learning, is defined as the learning that happens in the absence of a teacher or supervisor; it is achieved through adaptation to past environments, which is necessary for higher brain functions. An understanding of the physiological mechanisms that mediate unsupervised learning is fundamental to augmenting our knowledge of information processing in the brain.

One of the consequent benefits of unsupervised learning is inference, which is the action of guessing unknown matters based on known facts or certain observations, i.e., the process of drawing conclusions through reasoning and estimation. While inference is thought to be an act of the conscious mind in the ordinary sense of the word, it can occur even in the unconscious mind. Hermann von Helmholtz, a 19th-century physicist/physiologist, realized that perception often requires inference by the unconscious mind and coined the word *unconscious inference* [11]. According to Helmholtz, conscious inference and unconscious inference can be distinguished based on whether conscious knowledge is involved in the process. For example, when an astronomer computes the positions or distances of stars in space based on images taken at various times from different parts of the orbit of the Earth, he or she performs conscious inference because the process is “based on a conscious knowledge of the laws of optics”; by contrast, “in the ordinary acts of vision, this knowledge of optics is lacking” [11]. Thus, the latter process is performed by the unconscious mind. Unconscious inference is crucial for estimating the overall picture from partial observations.

In the field of theoretical and computational neuroscience, unconscious inference has been translated as the successive inference of the generative process of the external world (in terms of Bayesian inference) that animals perform in order to achieve perception. One hypothesis, the so-called internal model hypothesis [12,13,14,15,16,17,18,19], states that animals reconstruct a model of the external world in their brain through past experiences. This internal model helps animals infer hidden causes and predict future inputs automatically; in other words, this inference process happens unconsciously. This is also known as the predictive coding hypothesis [20,21]. In the past decade, a mathematical foundation for unconscious inference, called the free-energy principle (FEP), has been proposed [13,14,15,16,17], and is a candidate unified theory of higher brain functions. Briefly, this principle hypothesizes that parameters of the generative model are learned through unsupervised learning, while hidden variables are inferred in the subsequent inference step. The FEP provides a unified framework for higher brain functions including perceptual learning [14], reinforcement learning [22], motor learning [23,24], communication [25,26], emotion, mental disorders [27,28], and evolution. However, the difference between the FEP and a related theory, namely the information maximization (infomax) principle, which states that a neural network maximizes the amount of sensory information preserved in the network [29,30,31,32], is still not fully understood.

In this study, the relationship between the FEP and the infomax principle is investigated. As one of the most simple and important examples, the study focuses on blind source separation (BSS), which is the task of separating sensory inputs into hidden sources (or causes) [33,34,35,36]. BSS is shown to be a subset of the inference problem considered in the FEP, and variational free energy is demonstrated to represent the difference between the information stored in the neural network (which is the measure of the infomax principle [29]) and the information available for inferring current sensory inputs.

## 2. Methods

### 2.1. Definition of a System

Let us suppose $s\equiv {\left(s_{1},\cdots ,s_{N}\right)}^{T}$ as hidden sources that follow $p\left(s|\lambda \right)\equiv \prod_{i}p\left(s_{i}|\lambda \right)$ parameterized by a hyper-parameter set λ; $x\equiv {\left(x_{1},\cdots ,x_{M}\right)}^{T}$ as sensory inputs; $u\equiv {\left(u_{1},\cdots ,u_{N}\right)}^{T}$ as neural outputs; $z\equiv {\left(z_{1},\cdots ,z_{M}\right)}^{T}$ as background noises that follow p(z|λ) parameterized by λ; $\epsilon \equiv {\left(\epsilon_{1},\cdots ,\epsilon_{M}\right)}^{T}$ as reconstruction errors; and $f\in R^{M}$, $g\in R^{N}$, and $h\in R^{M}$ as nonlinear functions (see also Table 1). The generative process of the external world (or the environment) is described by a stochastic equation as:

(1) Generativeprocess:x=f(s)+z.

Recognition and generative models of the neural network are defined as follows:

(2) Recognitionmodel:u=g(x),

(3) Generativemodel:x=h(u)+ϵ.

**Figure 1:**
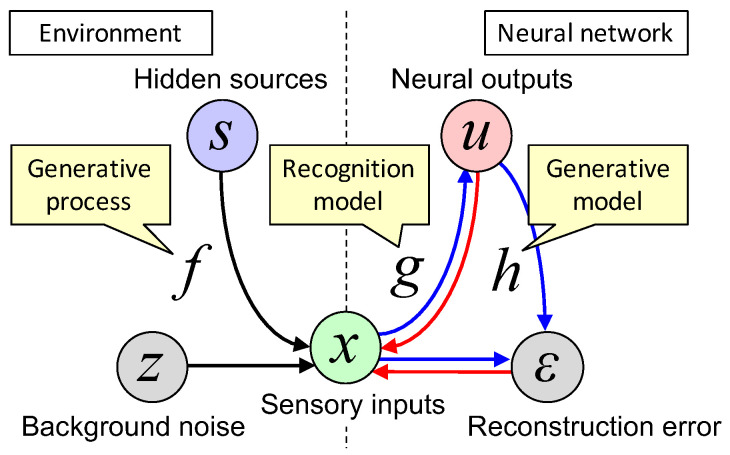
Schematic images of a generative process of the environment (left) and recognition and generative models of the neural network (right). Note that the neural network can access only the states in the right side of the dashed line, including *x* (see text in Section 2.2). Black arrows are causal relationships in the external world. Blue arrows are information flows of the neural network (i.e., actual causal relationships in the neural network), while red arrows are hypothesized causal relationships (to imitate the external world) when the generative model is considered. See main text and Table 1 for meanings of variables and functions.

Figure 1 illustrates the structure of the system under consideration. For the generative model, the prior distribution of *u* is defined as $p^{*}\left(u|\gamma \right)=\prod_{i}p^{*}\left(u_{i}|\gamma \right)$ with a hyper-parameter set γ and the likelihood function as $p^{*}\left(x|h\left(u\right),\gamma \right)=N\left[x;h\left(u\right),\Sigma_{\epsilon }\left(\gamma \right)\right]$, where $p^{*}$ indicates a statistical model and N is a Gaussian distribution characterized by the mean h(u) and covariance $\Sigma_{\epsilon }\left(\gamma \right)$. Moreover, suppose θ, $W\in R^{N\times M}$, and $V\in R^{M\times N}$ as parameter sets for *f*, *g*, and *h*, respectively, λ as a hyper-parameter set for p(s|λ) and p(z|λ), and γ as a hyper-parameter set for $p^{*}\left(u|\gamma \right)$ and $p^{*}\left(x|h\left(u\right),\gamma \right)$. Here, hyper-parameters are defined as parameters that determine the shape of distributions (e.g., the covariance matrix). Note that *W* and *V* are assumed as synaptic strength matrices for feedforward and backward paths, respectively, while γ is assumed as a state of neuromodulators similarly to [13,14,15]. In this study, unless specifically mentioned, parameters and hyper-parameters refer to slowly changing variables, so that *W*, *V*, and γ can change their values. Equations (1)–(3) are transformed into probabilistic representations.

(4) $$\begin{array}{cc} \mathrm{Generative}\;\mathrm{process}:\;\;p(s,x|\theta ,\lambda ) & =p(x|s,\theta ,\lambda )p(s|\lambda )\\ & =\int \delta (x-f(s;\theta )-z)p(z|\lambda )p(s|\lambda )dz\\ & =p(z=x-f|s,\theta ,\lambda )p(s|\lambda ), \end{array}$$

(5) $$\begin{array}{cc} \mathrm{Recognition}\;\mathrm{model}:\;\;p(x,u|W) & =p(x|u,W)p(u|W)\\ & =p(u|x,W)p(x)\\ & =p(u-g(x;W)|x,W)p(x), \end{array}$$

(6) $$\begin{array}{cc} \mathrm{Generative}\;\mathrm{model}:\;\;p^{*}\left(x,u|V,\gamma \right) & =p^{*}\left(x|u,V,\gamma \right)p^{*}\left(u|\gamma \right)\\ & =\int \delta \left(x-h\left(u;V\right)-\epsilon \right)p^{*}\left(\epsilon |V,\gamma \right)p^{*}\left(u|\gamma \right)d\epsilon \\ & =p^{*}\left(\epsilon =x-h|u,V,\gamma \right)p^{*}\left(u|\gamma \right). \end{array}$$

Note that δ(·) is Dirac’s delta function and $p^{*}\left(x|u,V,\gamma \right)\equiv p\left(x|u,V,\gamma ,m\right)$ is a statistical model given a model structure *m*. For simplification, let ϑ≡{s,θ,λ} be a set of hidden states of the external world and φ≡{u,W,V,γ} be a set of internal states of the neural network. By multiplying p(θ,λ) by Equation (4), p(W,V,γ) by Equation (5), and $p^{*}\left(W,V,\gamma \right)=p^{*}\left(W\right)p^{*}\left(V\right)p^{*}\left(\gamma \right)$ by Equation (6), Equations (4)–(6) become

(7) Generativeprocess:p(x,ϑ)=p(x|ϑ)p(ϑ)=p(z=x−f|ϑ)p(ϑ),

(8) Recognitionmodel:p(x,φ)=p(x|φ)p(φ)=p(ϵ=x−h|φ)p(φ),

(9) $$\mathrm{Generative}\;\mathrm{model}:\;\;p^{*}\left(x,\varphi \right)=p^{*}\left(x|\varphi \right)p^{*}\left(\varphi \right)=p^{*}\left(\epsilon =x-h|\varphi \right)p^{*}\left(\varphi \right),$$

where $p^{*}\left(\varphi \right)=p^{*}\left(u|\gamma \right)p^{*}\left(W,V,\gamma \right)$ is the prior distribution for φ and $p^{*}\left(x,\varphi \right)\equiv p\left(x,\varphi |m\right)$ is a statistical model given a model structure *m*, which is determined by the shapes of $p^{*}\left(\varphi \right)$ and $p^{*}\left(x|\varphi \right)\equiv p^{*}\left(x|u,V,\gamma \right)$. The expression of $p^{*}\left(x,\varphi \right)$ is used instead of p(x,φ|m) to emphasize the difference between p(x,φ) and $p^{*}\left(x,\varphi \right)$. While p(x,φ)≡p(u|x,W)p(W,V,γ|x)p(x) is the actual joint probability of (x,φ) (which corresponds to the posterior distribution), $p^{*}\left(x,\varphi \right)$, i.e., the product of the likelihood function and the prior distribution, represents the generative model that the neural network expects (x,φ) to follow. Typically, elements of $p^{*}\left(W,V,\gamma \right)$ are supposed to be independent of each other, $p^{*}\left(W,V,\gamma \right)=\prod_{ii^{\prime }}p^{*}\left(W_{ii^{\prime }}\right)\prod_{jj^{\prime }}p^{*}\left(V_{jj^{\prime }}\right)\prod_{k}p^{*}\left(\gamma_{k}\right)$. For example, sparse priors about parameters are sometimes used to prevent the over-learning [37], while a generative model with sparse priors for outputs is known as a sparse coding model [38,39]. As shown later, the inference and learning are achieved by minimizing the difference between p(x,φ) and $p^{*}\left(x,\varphi \right)$. At that time, minimizing the difference between p(V,W,γ) and $p^{*}\left(V,W,\gamma \right)$ acts as a constraint or a regularizer that prevents over-learning (see Section 2.3 for details).

### 2.2. Information Stored in the Neural Network

Information is defined as the negative log of probability [40]. When Prob(x) is the probability of given sensory inputs *x*, its information is given by −logProb(x) [nat], where 1 nat = 1.4427 bits. When *x* takes continuous values, by coarse graining, −logProb(x) is replaced with $-\log(p\left(x\right)\Delta_{x})$, where p(x) is the probability density of *x* and $\Delta_{x}\equiv \prod_{i}\Delta_{x_{i}}$ is the product of the finite spatial resolutions of *x*’s elements ($\Delta_{x_{i}}>0$). The expectation of $-\log(p\left(x\right)\Delta_{x})$ over p(x) gives the Shannon entropy (or average information), which is defined by

(10) $$H\left[x\right]\equiv {\langle -\log(p\left(x\right)\Delta_{x})\rangle }_{p(x)}\left[\mathrm{nat}\right],$$

where ${\langle \cdot \rangle }_{p(x)}\equiv \int \cdot p\left(x\right)dx$ represents the expectation of · over p(x). Note that the use of $-\log(p\left(x\right)\Delta_{x})$ instead of −logp(x) is useful because this H[x] is non-negative ($d\mathrm{Prob}\left(x\right)=\;p\left(x\right)\Delta_{x}$ takes a value between 0 and 1). This is a coarse binning of *x* and the spatial resolution $\Delta_{x}$ takes a small but nonzero value so that the addition of constant $-\log\Delta_{x}$ has no effect except for sliding the offset value. If and only if p(x) is Dirac’s delta function (strictly, $p\left(x\right)=1/\Delta_{x}$ at one bin and 0 otherwise), H[x]=0 is realized. For the system under consideration (Equations (7)–(9)), the information shared between the external world states (x,ϑ) and the internal states of the neural network φ is defined by mutual information [41]

(11) $$I\left[\left(x,\vartheta \right);\varphi \right]\equiv {\langle \log\frac{p(x,\vartheta ,\varphi )}{p(x,\vartheta )p(\varphi )}\rangle }_{p(x,\vartheta ,\varphi )}\left[\mathrm{nat}\right].$$

Note that p(x,ϑ,φ) is the joint probability of (x,ϑ) and φ. Moreover p(x,ϑ) and p(φ) are their marginal distributions, respectively. This mutual information takes a non-negative value and quantifies how much (x,ϑ) and φ are related with each other. High mutual information indicates the internal states are informative for explaining the external world states, while zero mutual information means they are independent of each other.

However, the only information that the neural network can directly access is the sensory input. This is the case because the system under consideration can be described as a Bayesian network (see [42,43] for details on the Markov blanket). Hence, the entropy of the external world states under a fixed sensory input gives information that the neural network cannot infer. Moreover, there is no feedback control from the neural network to the external world in this setup. Thus, under a fixed *x*, ϑ and φ are conditionally independent of each other. From p(ϑ,φ|x)=p(ϑ|x)p(φ|x), we can obtain

(12) $$\begin{array}{c} I\left[\left(x,\vartheta \right);\varphi \right]={\langle \log\frac{p(\vartheta |x)p(\varphi |x)p(x)}{p(\vartheta |x)p(x)p(\varphi )}\rangle }_{p(\vartheta |x)p(\varphi |x)p(x)}={\langle \log\frac{p(\varphi |x)}{p(\varphi )}\rangle }_{p(\varphi ,x)}=I\left[x;\varphi \right]. \end{array}$$

Using Shannon entropy, I[x;φ] becomes

(13) I[x;φ]=H[x]−H[x|φ][nat],

where

(14) $$H\left[x|\varphi \right]\equiv {\langle -\log\left(p\left(x|\varphi \right)\Delta_{x}\right)\rangle }_{p(x,\varphi )}$$

is the conditional entropy of *x* given φ. Thus, maximization of I[(x,ϑ);φ] is the same as maximization of I[x;φ] for this system. As I[x;φ], H[x], and H[x|φ] are non-negative, I[x;φ] has the range 0≤I[x;φ]≤H[x]. Zero mutual information occurs if and only if *x* and φ are independent, while I[x;φ]=H[x] occurs if and only if *x* is fully explained by φ. In this manner, I[x;φ] describes the information about the external world stored in the neural network. Note that this I[x;φ] can be expressed using the Kullback–Leibler divergence (KLD) [44] as $I\left[x;\varphi \right]\equiv D_{KL}\left[p(x,\varphi )||p(x)p(\varphi )\right]$. The KLD takes a non-negative value and indicates the divergence between two distributions.

The infomax principle states that “the network connections develop in such a way as to maximize the amount of information that is preserved when signals are transformed at each processing stage, subject to certain constraints” [29], see also [30,31,32]. According to the infomax principle, the neural network is hypothesized to maximize I[x;φ] to perceive the external world. However, I[x;φ] does not fully explain the inference capability of a neural network. For example, if neural outputs just express the sensory input itself (u=x), I[x;φ]=H[x] is easily achieved, but this does not mean that the neural network can predict or reconstruct input statistics. This is considered in the next section.

### 2.3. Free-Energy Principle

If one has a statistical model determined by model structure *m*, the information calculated based on *m* is given by the negative log likelihood −logp(x|m), which is termed as the surprise (or the marginal likelihood) of the sensory input and expresses the unpredictability of the sensory input for the individual. The neural network is considered to minimize the surprise in the sensory input using the knowledge about the external world, to perceive the external world [13]. To infer if an event is likely to happen based on the past observation, a statistical (i.e., generative) model is necessary; otherwise it is difficult to generalize sensory inputs [45]. Note that the surprise is the marginal over the generative model; hence, the neural network can reduce the surprise by optimizing its internal states, while Shannon entropy of the input is determined by the environment. When the actual probability density and a generative model are given by p(x) and $p^{*}\left(x\right)\equiv p\left(x|m\right)$, respectively, the cross entropy ${\langle -\log\left(p^{*}\left(x\right)\Delta_{x}\right)\rangle }_{p(x)}$ is always larger than or equal to Shannon entropy H[x] because of the non-negativity of KLD. Hence, in this study, the input surprise is defined by

(15) $$S\left(x\right)\equiv -\log p^{*}\left(x\right)+\log p\left(x\right)\;\;\left[\mathrm{nat}\right]$$

and its expectation over p(x) by

(16) $$\bar{S}\equiv {\langle S\left(x\right)\rangle }_{p(x)}=D_{KL}\left[p\left(x\right)|\right|p^{*}\left(x\right)]={\langle -\log(p^{*}\left(x\right)\Delta_{x})\rangle }_{p(x)}-H\left[x\right]\;\left[\mathrm{nat}\right].$$

This definition of S(x) is to ensure $\bar{S}$ is non-negative and $\bar{S}=0$ if and only if $p^{*}\left(x\right)=p\left(x\right)$. Since H[x] is determined by the environment and constant for the neural network, minimization of this $\bar{S}$ is the same meaning as minimization of ${\langle -\log(p^{*}\left(x\right)\Delta_{x})\rangle }_{p(x)}$.

As the sensory input is generated by the external world generative process, consideration of the structure and dynamics placed in the background of the sensory input can provide accurate inference. According to the internal model hypothesis, animals develop the internal model in their brain to increase the accuracy and efficiency of inference [12,13,14,15,17,18,19]; thus, internal states of the neural network φ are hypothesized to imitate the hidden states of the external world ϑ. A problem is that $-\log p^{*}\left(x\right)=-\log\left(\int p^{*}\left(x,\varphi \right)d\varphi \right)$ is intractable for the neural network, because the integral of $p^{*}\left(x,\varphi \right)$ placed in the logarithm function. The FEP hypothesizes that the neural network calculates an upper bound of $-\log p^{*}\left(x\right)$ instead of the exact value as a proxy, which is more tractable [13] (because −logp(x) is fixed, the free energy is sometimes defined including or excluding this term). This upper bound is termed as variational free energy:

(17) $$F\left(x\right)\equiv S\left(x\right)+D_{KL}\left[p\left(\varphi |x\right)|\right|p^{*}\left(\varphi |x\right)]={\langle -\log p^{*}\left(x,\varphi \right)+\log p\left(x,\varphi \right)\rangle }_{p(\varphi |x)}\left[\mathrm{nat}\right].$$

Note that p(φ|x)≡p(u|x,W)p(W,V,γ|x) expresses the belief about hidden states of the external world encoded by internal states of the neural network, termed as the recognition density. Due to the non-negativity of KLD, F(x) is guaranteed to be an upper bound of S(x) and F(x)=S(x) holds if and only if $p^{*}\left(\varphi |x\right)=p\left(\varphi |x\right)$. Furthermore, the expectation of F(x) over p(x) is defined by

(18) $$\begin{array}{cc} \bar{F} & \equiv {\langle F\left(x\right)\rangle }_{p(x)}=D_{KL}\left[p\left(x,\varphi \right)|\right|p^{*}\left(x,\varphi \right)]\\ & ={\langle -\log(p^{*}\left(x|\varphi \right)\Delta_{x})\rangle }_{p(x,\varphi )}+{\langle -\log(p^{*}\left(\varphi \right)\Delta_{\varphi })\rangle }_{p(\varphi )}-H\left[\varphi |x\right]-H\left[x\right]\;\left[\mathrm{nat}\right], \end{array}$$

where ${\langle -\log(p^{*}\left(x|\varphi \right)\Delta_{x})\rangle }_{p(x,\varphi )}$ is the negative log likelihood and called the accuracy [15]. The second and third terms are the cross entropy of φ and the conditional entropy of φ given *x*, $H\left[\varphi |x\right]\equiv {\langle -\log\left(p\left(\varphi |x\right)\Delta_{\varphi }\right)\rangle }_{p(x,\varphi )}$, where the difference between them is called the complexity [15]. The last term H[x] is a constant. $\bar{F}$ indicates the difference between the actual probability p(x,φ) and the generative model $p^{*}\left(x,\varphi \right)$. Given the non-negativity of KLD, $\bar{F}$ is always larger than or equal to non-negative value $\bar{S}$, and $\bar{F}=\bar{S}=0$ holds if and only if $p^{*}\left(x,\varphi \right)=p\left(x,\varphi \right)$. The FEP hypothesized that $\bar{F}$ is minimized by optimizing neural activities (*u*), synaptic strengths (*W* and *V*; i.e., synaptic plasticity), and activities of neuromodulators (γ).

The accuracy ${\langle -\log(p^{*}\left(x|\varphi \right)\Delta_{x})\rangle }_{p(x,\varphi )}$ quantifies the amplitude of the reconstruction error. Minimization of the accuracy is the maximum likelihood estimation [10] and provides a solution that (at least locally) minimizes the reconstruction error. Whereas, minimization of the complexity ${\langle -\log(p^{*}\left(\varphi \right)\Delta_{\varphi })\rangle }_{p(\varphi )}-H\left[\varphi |x\right]$ makes p(φ) closer to $p^{*}\left(\varphi \right)$. As $p^{*}\left(\varphi \right)=p^{*}\left(u|\gamma \right)p^{*}\left(W,V,\gamma \right)$ usually supposes the elements of φ are mutually independent, this acts as the maximization of the entropy under a constraint. Hence, this leads to the increase of the independence between internal states, which helps neurons to establish an efficient representation, as pointed out by Jaynes’ max entropy principle [46,47]. This is essential for BSS [33,34,35,36] because the optimal parameters that minimize the accuracy are not always uniquely determined. Due to this, the maximum likelihood estimation alone does not always identify the generative process behind the sensory inputs. As $\bar{F}$ is the sum of costs for the maximum likelihood estimation and BSS, free-energy minimization is the rule to simultaneously minimize the reconstruction error and maximize the independence of the internal states. It is recognized that animals perform BSS [2,3,4,5,6,7]. Interestingly, even *in vitro* neural networks perform BSS, which is accompanied by significant reduction of free energy in accordance with the FEP and Jaynes’ max entropy principle [48].

### 2.4. Information Available for Inference

We now consider how free energy expectation $\bar{F}$ relates to mutual information I[x;φ]. According to unconscious inference and the internal model hypothesis, the aim of a neural network is to predict *x*, and for this purpose, it infers hidden states of the external world. While the neural network is conventionally hypothesized to express sufficient statistics of the hidden states of the external world [14], here it is hypothesized that internal states of the neural network are random variables and the probability distribution of them imitates the probability distribution of the hidden states of the external world. The neural network hence attempts to match the joint probability of the sensory inputs and the internal states with that of the sensory inputs and the hidden states of the external world. To do so, the neural network shifts the actual probability of internal states p(x,φ)=p(x|φ)p(φ) closer to those of the generative model $p^{*}\left(x,\varphi \right)=p^{*}\left(x|\varphi \right)p^{*}\left(\varphi \right)$ that the neural network expects (x,φ) to follow (note that here, p(x|φ)=p(x|u,W) and $p^{*}\left(x|\varphi \right)=p^{*}\left(x|u,V,\gamma \right)$). This means that the shape or structure of $p^{*}\left(x,\varphi \right)$ is pre-defined, but the argument (x,φ) can still change. From this viewpoint, the difference between these two distributions is associated with the loss of information.

**Figure 2:**
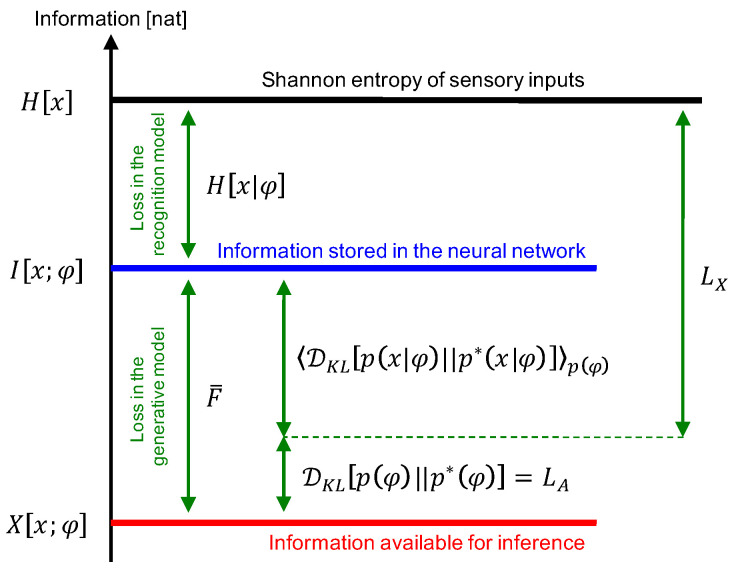
Relationship between information measures. The mutual information between the inputs and internal states of the neural network (I[x;φ]) is less than or equal to the Shannon entropy of the inputs (H[x]) because of the information loss in the recognition model. The utilizable information (X[x;φ]) is less than or equal to the mutual information, and the gap between them gives the expectation of the variational free energy ($\bar{F}$), which quantifies the loss in the generative model. The sum of the principal component analysis (PCA) and independent component analysis (ICA) costs ($L_{X}+L_{A}$) is equal to the gap between the Shannon entropy and the utilizable information, expressing the sum of losses in the recognition and generative models.

The amount of information available for inference can be calculated using the following three values related to information loss: (i) because H[x] is information of the sensory input and I[x;φ] is information stored in the neural network, H[x]−I[x;φ]=H[x|φ] indicates the information loss in the recognition model (Figure 2); (ii) the difference between actual and desired (prior) distributions of internal states $D_{KL}\left[p\left(\varphi \right)|\right|p^{*}\left(\varphi \right)]$ quantifies the information loss for inferring internal states using the prior (i.e., blind state separation). This is a common approach used in BSS methods [33,34,35,36]; and (iii) the difference between distributions of the actual reconstruction error and the reconstruction error under the given model $\langle D_{KL}\left[p\left(x|\varphi \right)|\right|p^{*}\left(x|\varphi \right){]\rangle }_{p(\varphi )}$ quantifies the information loss for representing inputs using internal states. Therefore, by subtracting these three values from H[x], a mutual-information-like measure representing the inference capability is obtained:

(19) $$\begin{array}{cc} X[x;\varphi ] & \equiv H\left[x\right]-H\left[x|\varphi \right]-D_{KL}\left[p\left(\varphi \right)|\right|p^{*}\left(\varphi \right)]-\langle D_{KL}\left[p\left(x|\varphi \right)|\right|p^{*}\left(x|\varphi \right){]\rangle }_{p(\varphi )}\\ & ={\langle \log\frac{p^{*}\left(x,\varphi \right)}{p(x)p(\varphi )}\rangle }_{p(x,\varphi )}\;\left[\mathrm{nat}\right], \end{array}$$

which is called utilizable information in this study. This utilizable information X[x;φ] is defined by replacing p(x,φ) in I[x;φ] with $p^{*}\left(x,\varphi \right)$, immediately yielding

(20) $$\bar{F}=I\left[x;\varphi \right]-X\left[x;\varphi \right]\;\left[\mathrm{nat}\right].$$

Hence, $\bar{F}$ represents the gap between the amount of information stored in the neural network and the amount that is available for inference, which is equivalent to the information loss in the generative model. Note that the sum of losses in the recognition and generative models $H\left[x\right]-X\left[x;\varphi \right]=\bar{F}+H\left[x|\varphi \right]$ is an upper bound of $\bar{F}$ because of the non-negativity of H[x|φ] (Figure 2). As H[x|φ] is generally nonzero, F(x)+H[x|φ] does not usually reach zero, even when $p\left(x,\varphi \right)=p^{*}\left(x,\varphi \right)$.

Furthermore, X[x;φ] is transformed into

(21) $$X\left[x;\varphi \right]=H\left[x\right]-L_{X}-L_{A},$$

where

(22) $$L_{X}\equiv {\langle -\log(p^{*}\left(x|\varphi \right)\Delta_{x})\rangle }_{p(x,\varphi )}$$

is the so-called reconstruction error, which is similar to the reconstruction error for principal component analysis (PCA) [49], while

(23) $$L_{A}\equiv D_{KL}\left[p\left(\varphi \right)|\right|p^{*}\left(\varphi \right)]$$

is a generalization of Amari’s cost function for independent component analysis (ICA) [50].

PCA is one of the most popular dimensionality reduction methods. It is used to remove background noise and extract important features from sensory inputs [49,51]. In contrast, ICA is a BSS method used to decompose a mixture set of sensory inputs into independent hidden sources [34,36,50,52,53]. Theoreticians hypothesize that the PCA- and ICA-like learning underlies BSS in the brain [3]. This kind of extraction of the hidden representation is also an important problem in machine learning [54,55]. Equation (21) indicates that X[x;φ] consists of PCA- and ICA-like parts, i.e., maximization of X[x;φ] can perform both dimensionality reduction and BSS (Figure 2). Their relationship is discussed in the next section.

## 3. Comparison between the Free-Energy Principle and Related Theories

In this section, the FEP is compared with other theories. As described in the Methods, the aim of the infomax principle is to maximize mutual information I[x;φ] (Equation (13)), while the aim of the FEP is to minimize free energy expectation $\bar{F}$ (Equation (18)), while maximization of utilizable information X[x;φ] (Equation (19)) means to do both of them simultaneously.

### 3.1. Infomax Principle

The generative process and the recognition and generative models defined in Equations (1)–(3) are assumed. For the sake of simplicity, let us suppose W,V, and γ follow Dirac’s delta functions; then, the goal of the infomax principle is simplified to maximization of the mutual information between the sensory inputs *x* and the neural outputs *u*:

(24) $$I\left[x;u|W\right]={\langle \log\frac{p(x,u|W)}{p(x)p(u|W)}\rangle }_{p(x,u,W)}=H\left[x\right]-H\left[x|u,W\right]=H\left[u|W\right]-H\left[u|x,W\right].$$

Here *W*, *V*, and γ are still variables, and *W* is optimized according to the learning while *V* and γ do not directly contribute to minimization of I[x;u|W]. For the sake of simplicity, let us suppose dim(x)≥dim(u) and a linear recognition model u=g(x)=Wx, with full-rank matrix *W*. As H[u|x,W]=const. is usually assumed and *u* has an infinite range, I[x;u|W]=H[u|W]+const. monotonically increases as the variance of *u* increases. Thus, I[x;u|W] without any constraint is insufficient for deriving learning algorithms for PCA or ICA. To perform PCA and ICA based on the infomax principle, one may consider mutual information between the sensory inputs and the nonlinearly transformed neural outputs $\psi \left(u\right)={\left(\psi \left(u_{1}\right),\cdots ,\psi \left(u_{N}\right)\right)}^{T}$ with an injective nonlinear function ψ(·). This mutual information is given by:

(25) $$I\left[x;\psi \left(u\right)|W\right]={\langle \log\frac{p(x,\psi (u)|W)}{p(x)p(\psi (u)|W)}\rangle }_{p(x,\psi (u),W)}=H\left[\psi \left(u\right)|W\right]-H\left[\psi \left(u\right)|x,W\right].$$

When nonlinear neural outputs have a finite range (e.g., between 0 and 1), the variance of *u* should be maintained in the appropriate range. The infomax-based ICA [52,53] is formulated based on this constraint. From $p\left(\psi \left(u\right)|W\right)=|\partial u/\partial \psi \left(u\right)|p\left(u|W\right)={\left(\prod_{i}\psi^{\prime }\left(u_{i}\right)\right)}^{-1}p\left(u|W\right)$, H[ψ(u)|W] becomes $H\left[\psi \left(u\right)|W\right]={\langle -\log\left\{{\left(\prod_{i}\psi^{\prime }\left(u_{i}\right)\right)}^{-1}p\left(u|W\right)\Delta_{u}\right\}\rangle }_{p(u,W)}=H\left[u|W\right]+{\langle \sum_{i}\log\psi^{\prime }\left(u_{i}\right)\rangle }_{p(u,W)}$. Since H[ψ(u)|x,W]=const. holds, Equation (25) becomes:

(26) $$I\left[x;\psi \left(u\right)|W\right]=H\left[u|W\right]+{\langle \sum_{i}\log\psi^{\prime }\left(u_{i}\right)\rangle }_{p(u,W)}+\mathrm{const}.$$

This is the cost function that is usually considered in the studies on the infomax-based ICA [52,53]. The following section shows that PCA and ICA are performed by the maximization of Equation (26) as well as the FEP.

### 3.2. Principal Component Analysis

Both the infomax principle and FEP yield a cost function of PCA. One of the most popular data compression methods, PCA is defined by minimization of the error when the inputs are reconstructed from the compressed representation (i.e., *u* in this study) [49]. It is known that PCA is derived from the infomax principle under a constraint on the internal states. Although maximization of the mutual information between *x* and *u* under the orthonormal constraint on *W* is usually considered [29], here let us consider another solution. Suppose dim(x)>dim(u), $V=W^{T}$, and $\log\psi^{\prime }\left(u_{i}\right)=u_{i}^{2}/2+\mathrm{const}$. From Equation (24), H[u|W]=H[x]−H[x|u,W]+const. holds. Since the reconstruction error is given by $\epsilon =x-W^{T}u=\left(I-W^{T}W\right)x$ for the linear system under consideration, we obtain $H\left[x|u,W\right]=\langle -\log\{p\left(x\right)|\partial x/\partial \epsilon |\Delta_{x}{\}\rangle }_{p(x,\varphi )}=H\left[x\right]+\langle \log|I-W^{T}W{|\rangle }_{p(\varphi )}$. Thus, Equation (26) becomes:

(27) $$I\left[x;\psi \left(u\right)|W\right]={\langle -\log|I-W^{T}W|+\frac{1}{2}{\left|u\right|}^{2}\rangle }_{p(x,\varphi )}+\mathrm{const}.$$

The first term of Equation (27) is maximized if $WW^{T}=I$ holds (i.e., if *W* is an orthogonal matrix; here, a coarse graining with a finite resolution of *W* is supposed). To maximize the second term, outputs *u* need to be involved in a subspace spanned by the first to the *N*-th major principal components of *x*. Therefore, maximization of Equation (27) performs PCA.

Further, PCA is also derived by minimization of $L_{X}$ (Equation (22)), under the assumption that the reconstruction error follows a Gaussian distribution $p^{*}\left(x|\varphi \right)=p^{*}\left(x|u,W,V,\gamma \right)=N\left[x;W^{T}u,\gamma^{-1}I\right]$. Here, γ>0 is a scalar hyper-parameter that scales the precision of the reconstruction error. Hence, the cost function is given by:

(28) $$L_{X}={\langle \frac{\gamma }{2}\epsilon^{T}\epsilon -\frac{1}{2}\log\left|\gamma \right|\rangle }_{p(\varphi )}+\mathrm{const}.$$

When γ is fixed, the derivative of Equation (28) with respect to *W* gives the update rule for the least mean square error PCA [49]. As this cost function quantifies the magnitude of the reconstruction error, the algorithm that minimizes Equation (28) yields the low-dimensional compressed representation that minimizes the loss incurred in reconstructing the sensory inputs. This algorithm is the same as Oja’s subspace rule [51], up to an additional term that does not essentially change its behavior (see, e.g., [56] for a comparison between them). The $L_{X}$ here is also in the same form as the cost function for an auto-encoder [54].

Moreover, when the priors of u,W,V, and γ are flat, ${\langle -\log p^{*}\left(u|W\right)\rangle }_{p(u,W)}$ and $D_{KL}\left[p\left(W,V,\gamma \right)|\right|p^{*}\left(W,V,\gamma \right)]$ are constants with respect to *u*, *W*, *V*, and γ, because p(W,V,γ) is supposed to be a delta function. Hence, the free energy expectation (Equation (18)) becomes $\bar{F}=L_{X}-H\left[x|\varphi \right]-H\left[u|W\right]=L_{X}+\mathrm{const}.$, where const. is a constant with respect to *u*, *W*, and *V*. In this case, the optimization of *W* gives the minimum of $\bar{F}$ because *u* and *V* are determined by *W* while γ is fixed. Thus, under this condition, $\bar{F}$ is equivalent to the cost function of the least mean square error PCA.

### 3.3. Independent Component Analysis

It is known that ICA yields independent representation of input data by maximizing the independence between the outputs [52,53]. Thus, ICA reduces the redundancy and yields an efficient representation. When sensory inputs are generated from hidden sources, representing the hidden sources is usually the most efficient representation. Both the infomax principle and FEP yield a cost function of ICA. Let us suppose that sources $s_{1},\cdots ,s_{N}$ independently follow an identical distribution $p_{0}\left(s_{i}|\lambda \right)$. The infomax-based ICA is derived from Equation (26) [52,53]. If $\psi (u_{i})$ is defined to satisfy $\psi^{\prime }\left(u_{i}\right)=p_{0}\left(u_{i}|\gamma \right)$, negative mutual information −I[x;ψ(u)|W] becomes the KLD between the actual and prior distributions up to a constant term,

(29) $$-I\left[x;\psi \left(u\right)|W\right]+\mathrm{const}.={\langle \log p\left(u|W\right)-\log p_{0}\left(u|\gamma \right)\rangle }_{p(\varphi )}={\langle D_{KL}\left[p\left(u|W\right)|\right|p_{0}\left(u|\gamma \right)]\rangle }_{p(W,V,\gamma )}=L_{A}.$$

The $L_{A}$ here is known as Amari’s ICA cost function [50], which is a reduction of (23). While both −I[x;ψ(u)|W] and $L_{A}$ provide the same gradient descent rule, formulating I[x;ψ(u)|W] requires nonlinearly transformed neural outputs ψ(u). By contrast, $L_{A}$ straightforwardly represents that ICA is performed by minimization of the KLD between p(u|W) and $p^{*}\left(u|\gamma \right)=p_{0}\left(u|\gamma \right)$. Indeed, if dim(u)=dim(x)=N, the background noise is small, and the priors of W,V, and γ are flat, we obtain $\bar{F}={\langle D_{KL}\left[p\left(u|W\right)|\right|p^{*}\left(u|\gamma \right)]\rangle }_{p(W,V,\gamma )}=L_{A}$. Therefore, ICA is a subset of the inference problem considered in the FEP, and the derivation from the FEP is simpler, although both the infomax principle and FEP yield the same ICA algorithm.

Furthermore, when dim(x)>dim(u), minimization of $\bar{F}$ can perform both dimensionality reduction and BSS. When the priors of W,V, and γ are flat, free energy expectation (Equation (18)) approximately becomes $\bar{F}\approx L_{X}+L_{A}+\mathrm{const}.=-X\left[x;u|W,V,\gamma \right]+\mathrm{const}$. Here, γ is fixed so that const. is a constant with respect to x,u,W and *V*. Conditional entropy H[x|u,W] is ignored in the calculation because it is typically of a smaller order than $L_{X}$ when Σ(γ) is not fine-tuned. As γ parameterizes the precision of the reconstruction error, it controls the ratio of PCA to ICA. Hence, as γ decreases to zero, the solution shifts from a PCA-like to an ICA-like solution.

Unlike the case with the scalar γ described above, if $\Sigma_{\epsilon }\left(\gamma \right)$ is fine-tuned by high-dimensional γ to minimize $\bar{F}$, $\Sigma_{\epsilon }={\langle \epsilon \epsilon^{T}\rangle }_{p(x,\varphi )}$ is obtained. Under this condition, $L_{X}$ is equal to H[x|u,W] up to a constant term, and thereby, $\bar{F}=L_{A}+\mathrm{const}.$ is obtained. This indicates that $\bar{F}$ consists only of the ICA part. These comparisons suggest that low-dimensional γ is better for performing noise reduction than high-dimensional γ.

## 4. Simulation and Results

**Figure 3:**
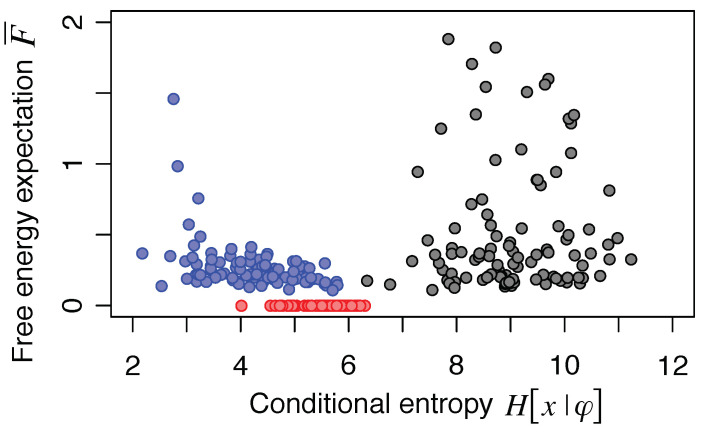
Difference between the infomax principle and free-energy principle (FEP) when sources follow a non-Gaussian distribution. Black, blue, and red circles indicate the results when *W* is a random matrix, optimized for the infomax principle (i.e., PCA), and optimized for the FEP, respectively.

The difference between the infomax principle and the FEP is illustrated by a simple simulation using a linear generative process and a linear neural network (Figure 3). For simplification, it is assumed that *u* quickly converge to u=Wx compared to the change of *s* (adiabatic approximation).

For the results shown in Figure 3, *s* denotes two-dimensional hidden sources following an identical Laplace distribution with zero mean and unit variance; *x* denotes four-dimensional sensory inputs; *u* denotes two-dimensional neural outputs; *z* denotes four-dimensional background Gaussian noises following $N[z;0,\Sigma_{z}]$; θ denotes a 4×2-dimensional mixing matrix; *W* is a 2×4-dimensional synaptic strength matrix for the bottom-up path; and *V* is a 4×2-dimensional synaptic strength matrix for the top-down path. The priors of W,V, and γ are supposed to be flat as in Section 3. Sensory inputs are determined by x=θs+z, while neural outputs are determined by u=Wx. The reconstruction error is given by ϵ=x−Vu and used to calculate H[x|φ] and $L_{A}$. Horizontal and vertical axes in the figure are conditional entropy H[x|φ] (Equation (14)) and free energy expectation $\bar{F}$ (Equation (18)), respectively. Simulations were conducted 100 times with randomly selected θ and $\Sigma_{z}$ for each condition. For each simulation, $10^{8}$ random sample points were generated and probability distributions were calculated using the histogram method.

First, when *W* is randomly chosen and *V* is defined by $V=W^{T}$, both H[x|φ] and $\bar{F}$ are scattered (black circles in Figure 3) because neural outputs represent random mixtures of sources and noises. Next, when *W* is optimized according to either Equation (27) or (28) under the constraint of $V=W^{T}$, the neural outputs express the major principal components of the inputs, i.e., the network performs PCA (blue circles in Figure 3). This is the case when H[x|φ] is minimized. In contrast, when W,V, and $\Sigma_{\epsilon }\left(\gamma \right)$ are optimized according to the FEP (see Equation (18)), the neural outputs represent the independent components that match the prior source distribution; i.e., the network performs BSS or ICA while reducing the reconstruction error (red circles in Figure 3). For linear generative processes, the minimization of $\bar{F}$ can reliably and accurately perform both dimensionality reduction and BSS because the outputs become independent of each other and match the prior belief if and only if the outputs represent true sources up to permutation and sign-flip. As the utilizable information consists of PCA and ICA cost functions (see Equation (21)), the maximization of X[x;φ] leads to a solution that is a compromise between the solutions for the infomax principle and the FEP. Interestingly, the infomax optimization (i.e., PCA) provides a *W* that makes $\bar{F}$ closer to zero than random states, which indicates that the infomax optimization contributes to the free energy minimization. Note that, for nonlinear systems, there are many different transformations that make the outputs independent of each other [57]. Hence, there is no guarantee that minimization of $\bar{F}$ can identify the true sources of nonlinear generative models.

In summary, the aims of the FEP and infomax principle are similar to each other. In particular, when both the sources and noises follow Gaussian distributions, their aims become the same. Conversely, the optimal synaptic weights under the FEP are different from those under the infomax principle when sources follow non-Gaussian distributions. Under this condition, the maximization of the utilizable information leads to a compromise solution between those for the FEP and the infomax principle.

## 5. Discussion

In this study, the FEP is linked with the infomax principle, PCA, and ICA. It is more likely that the purpose of a neural network in a biological system is to minimize the surprise of sensory inputs to realize better inference rather than maximize the amount of stored information. For example, the visual input captured by a video camera contributes to the stored information, but this amount of information is not equal to the amount of information available for inference. The surprise expectation represents the difference between actual and inferred observations; the free energy expectation provides the difference between recognition and generative models. Utilizable information is introduced to quantify the inference and generalization capability of sensory inputs. Using this approach, the free energy expectation can be explained as the gap between the information stored in the neural network and that available for inference.

To perform ICA based on the infomax principle, one needs to tune the nonlinearity of the neural outputs to ensure the derivative of the nonlinear I/O function matches the prior distribution. Conversely, under the FEP, ICA is straightforwardly derived from the KLD between the actual probability distribution and the prior distribution of *u*. Especially, in the absence of background noise and prior knowledge of the parameters and hyper-parameters, the free energy expectation is equivalent to the surprise expectation as well as Amari’s ICA cost function, which indicates that ICA is a subproblem of the FEP.

The variational free energy quantifies the gap between the actual probability and the generative model and is a straightforward extension of the cost functions for BSS in the sense that it comprises the cost function for PCA [49] and ICA [50] in some special cases. Apart from that, there are studies that use the gap between the actual probability and the product of the marginal distributions to perform BSS [58] or to evaluate the information loss [59,60]. While the relationship between the product of the marginal distributions and the generative model is non-trivial, the comparison would lead to a deeper understanding about how the information of the external world is encoded by the neural network. In the subsequent work, we would like to see how the FEP and the infomax principle are related to those approaches.

The FEP is a rigorous and promising theory from theoretical and engineering viewpoints because various learning rules are derived from the FEP [14,15]. However, to be a physiologically plausible theory of the brain, the FEP needs to satisfy certain physiological requirements. There are two major requirements: first, physiological evidence that shows the existence of learning or self-organizing processes under the FEP is required. The model structure under the FEP is consistent with the structure of cortical microcircuits [19]. Moreover, *in vitro* neural networks performing BSS reduce free energy [48]. It is known that the spontaneous prior activity of a visual area enables it to learn the properties of natural pictures [61]. These results suggest the physiological plausibility of the FEP. Nevertheless, further experiments and consideration of information-theoretical optimization under physiological constraints [62] are required to prove the existence of the FEP in the biological brain. Second, the update rule must be a biologically plausible local learning rule, i.e., synaptic strengths must be changed by signals from connected cells or widespread liquid factors. While the synaptic update rule for a discrete system is local [17], the current rule for a continuous system [14] is a non-local rule. Recently developed biologically-plausible three-factor learning models in which Hebbian learning is mediated by a third modulatory factor [56,63,64,65] may help reveal the neuronal mechanism underlying unconscious inference. Therefore, it is necessary to investigate how actual neural networks infer the dynamics placed in the background of the sensory input and whether this is consistent with the FEP (see also [66] for the relationship between the FEP and spike-timing dependent plasticity [67,68]). This may help develop a biologically plausible learning algorithm through which an actual neural network might develop its internal model. Characterization of information from a physical viewpoint may also help understand how the brain physically embodies the information [69,70]. In the subsequent work, we would like to investigate this relationship.

In summary, this study investigated the differences between two types of information: information stored in the neural network and information available for inference. It was demonstrated that free energy represents the gap between these two types of information. This result clarifies the difference between the FEP and related theories and can be utilized for understanding unconscious inference from a theoretical viewpoint.

## Figures and Tables

**Table 1:** Glossary of expressions.

Expression	Description
Generative process	A set of stochastic equations that generate the external world dynamics
Recognition model	A model in the neural network that imitates the inverse of the generative process
Generative model	A model in the neural network that imitates the generative process
$s\in R^{N}$	Hidden sources
$x\in R^{M}$	Sensory inputs
θ	A set of parameters
λ	A set of hyper-parameters
ϑ≡{s,θ,λ}	A set of hidden states of the external world
$u\in R^{N}$	Neural outputs
$W\in R^{N\times M},V\in R^{M\times N}$	Synaptic strength matrices
γ	State of neuromodulators
φ≡{u,W,V,γ}	A set of the internal states of the neural network
$z\in R^{M}$	Background noises
$\epsilon \in R^{M}$	Reconstruction errors
p(x)	The actual probability density of *x*
p(φ|x),p(x,φ),p(φ)	Actual probability densities (posterior densities)
$p^{*}\left(u|\gamma \right),p^{*}\left(\varphi \right)\equiv p^{*}\left(u|\gamma \right)p^{*}\left(W,V,\gamma \right)$	Prior densities
$p^{*}\left(x|\varphi \right)\equiv p^{*}\left(x|u,V,\gamma \right)$	Likelihood function
$p^{*}\left(x\right),p^{*}\left(\varphi |x\right),p^{*}\left(x,\varphi \right)$	Statistical models
$\Delta_{x}\equiv \prod_{i}\Delta_{x_{i}}$	Finite spatial resolution of *x*, $\Delta_{x_{i}}>0$
${\langle \cdot \rangle }_{p(x)}\equiv \int \cdot p\left(x\right)dx$	Expectation of · over p(x)
$H\left[x\right]\equiv {\langle -\log\left(p\left(x\right)\Delta_{x}\right)\rangle }_{p(x)}$	Shannon entropy of $p\left(x\right)\Delta_{x}$
${\langle -\log\left(p^{*}\left(x\right)\Delta_{x}\right)\rangle }_{p(x)}$	Cross entropy of $p^{*}\left(x\right)\Delta_{x}$ over p(x)
$D_{KL}\left[p\left(\cdot \right)|\right|p^{*}\left(\cdot \right)]\equiv {\langle \log\frac{p(\cdot )}{p^{*}\left(\cdot \right)}\rangle }_{p(\cdot )}$	KLD between p(·) and $p^{*}\left(\cdot \right)$
$I\left[x;\varphi \right]\equiv D_{KL}\left[p\left(x,\varphi \right)||p\left(x\right)p\left(\varphi \right)\right]$	Mutual information between *x* and φ
$S\left(x\right)\equiv \log\frac{p(x)}{p^{*}\left(x\right)}$	Surprise
$\bar{S}\equiv {\langle S\left(x\right)\rangle }_{p(x)}$	Surprise expectation
$F\left(x\right)\equiv S\left(x\right)+D_{KL}\left[p\left(\varphi |x\right)|\right|p^{*}\left(\varphi |x\right)]$	Free energy
$\bar{F}\equiv {\langle F\left(x\right)\rangle }_{p(x)}$	Free energy expectation
$X\left[x;\varphi \right]\equiv {\langle \log\frac{p^{*}\left(x,\varphi \right)}{p(x)p(\varphi )}\rangle }_{p(x,\varphi )}$	Utilizable information between *x* and φ

## References

[1] DiCarlo J.J., Zoccolan D., Rust N.C. (2012). How does the brain solve visual object recognition?. Neuron.

[2] Bronkhorst A.W. (2000). The cocktail party phenomenon: A review of research on speech intelligibility in multiple-talker conditions. Acta Acust. United Acust..

[3] Brown G.D., Yamada S., Sejnowski T.J. (2001). Independent component analysis at the neural cocktail party. Trends Neurosci..

[4] Haykin S., Chen Z. (2005). The cocktail party problem. Neural Comput..

[5] Narayan R., Best V., Ozmeral E., McClaine E., Dent M., Shinn-Cunningham B., Sen K. (2007). Cortical interference effects in the cocktail party problem. Nat. Neurosci..

[6] Mesgarani N., Chang E.F. (2012). Selective cortical representation of attended speaker in multi-talker speech perception. Nature.

[7] Golumbic E.M.Z., Ding N., Bickel S., Lakatos P., Schevon C.A., McKhann G.M., Schroeder C.E. (2013). Mechanisms underlying selective neuronal tracking of attended speech at a “cocktail party”. Neuron.

[8] Dayan P., Abbott L.F. (2001). Theoretical Neuroscience: Computational and Mathematical Modeling of Neural Systems.

[9] Gerstner W., Kistler W. (2002). Spiking Neuron Models: Single Neurons, Populations, Plasticity.

[10] Bishop C.M. (2006). Pattern Recognition and Machine Learning.

[11] Von Helmholtz H. (1962). Concerning the perceptions in general. Treatise on Physiological Optics.

[12] Dayan P., Hinton G.E., Neal R.M., Zemel R.S. (1995). The helmholtz machine. Neural Comput..

[13] Friston K., Kilner J., Harrison L. (2006). A free energy principle for the brain. J. Physiol. Paris.

[14] Friston K.J. (2008). Hierarchical model in the brain. PLoS Comput. Biol..

[15] Friston K. (2010). The free-energy principle: A unified brain theory?. Nat. Rev. Neurosci..

[16] Friston K. (2012). A free energy principle for biological systems. Entropy.

[17] Friston K., FitzGerald T., Rigoli F., Schwartenbeck P., Pezzulo G. (2017). Active inference: A process theory. Neural Comput..

[18] George D., Hawkins J. (2009). Towards a mathematical theory of cortical micro-circuits. PLoS Comput. Biol..

[19] Bastos A.M., Usrey W.M., Adams R.A., Mangun G.R., Fries P., Friston K.J. (2012). Canonical microcircuits for predictive coding. Neuron.

[20] Rao R.P., Ballard D.H. (1999). Predictive coding in the visual cortex: A functional interpretation of some extra-classical receptive-field effects. Nat. Neurosci..

[21] Friston K. (2005). A theory of cortical responses. Philos. Trans. R. Soc. Lond. B Biol. Sci..

[22] Friston K.J., Daunizeau J., Kiebel S.J. (2009). Reinforcement learning or active inference?. PLoS ONE.

[23] Kilner J.M., Friston K.J., Frith C.D. (2007). Predictive coding: An account of the mirror neuron system. Cognit. Process..

[24] Friston K., Mattout J., Kilner J. (2011). Action understanding and active inference. Biol. Cybern..

[25] Friston K.J., Frith C.D. (2015). Active inference, communication and hermeneutics. Cortex.

[26] Friston K., Frith C. (2015). A duet for one. Conscious. Cognit..

[27] Fletcher P.C., Frith C.D. (2009). Perceiving is believing: A Bayesian approach to explaining the positive symptoms of schizophrenia. Nat. Rev. Neurosci..

[28] Friston K.J., Stephan K.E., Montague R., Dolan R.J. (2014). Computational psychiatry: The brain as a phantastic organ. Lancet Psychiatry.

[29] Linsker R. (1988). Self-organization in a perceptual network. Computer.

[30] Linsker R. (1992). Local synaptic learning rules suffice to maximize mutual information in a linear network. Neural Comput..

[31] Lee T.W., Girolami M., Bell A.J., Sejnowski T.J. (2000). A unifying information-theoretic framework for independent component analysis. Comput. Math. Appl..

[32] Simoncelli E.P., Olshausen B.A. (2001). Natural image statistics and neural representation. Ann. Rev. Neurosci..

[33] Belouchrani A., Abed-Meraim K., Cardoso J.F., Moulines E. (1997). A blind source separation technique using second-order statistics. Signal Process. IEEE Trans..

[34] Choi S., Cichocki A., Park H.M., Lee S.Y. (2005). Blind source separation and independent component analysis: A review. Neural Inf. Process. Lett. Rev..

[35] Cichocki A., Zdunek R., Phan A.H., Amari S.I. (2009). Nonnegative Matrix and Tensor Factorizations: Applications to Exploratory Multi-Way Data Analysis and Blind Source Separation.

[36] Comon P., Jutten C. (2010). Handbook of Blind Source Separation: Independent Component Analysis and Applications.

[37] Palmer J., Rao B.D., Wipf D.P. (2004). Perspectives on sparse Bayesian learning. Adv. Neural Inf. Proc. Syst..

[38] Olshausen B.A., Field D.J. (1996). Emergence of simple-cell receptive field properties by learning a sparse code for natural images. Nature.

[39] Olshausen B.A., Field D.J. (1997). Sparse coding with an overcomplete basis set: A strategy employed by V1?. Vis. Res..

[40] Shannon C.E., Weaver W. (1949). The Mathematical Theory of Communication.

[41] Cover T.M., Thomas J.A. (1991). Elements of Information Theory.

[42] Pearl J. (1988). Probabilistic Reasoning in Intelligent Systems: Networks of Plausible Inference.

[43] Friston K.J. (2013). Life as we know it. J. R. Soc. Interface.

[44] Kullback S., Leibler R.A. (1951). On information and sufficiency. Ann. Math. Stat..

[45] Arora S., Risteski A. (2017). Provable benefits of representation learning. arXiv.

[46] Jaynes E.T. (1957). Information theory and statistical mechanics. Phys. Rev..

[47] Jaynes E.T. (1957). Information theory and statistical mechanics. II. Phys. Rev..

[48] Isomura T., Kotani K., Jimbo Y. (2015). Cultured Cortical Neurons Can Perform Blind Source Separation According to the Free-Energy Principle. PLoS Comput. Biol..

[49] Xu L. (1993). Least mean square error reconstruction principle for self-organizing neural-nets. Neural Netw..

[50] Amari S.I., Cichocki A., Yang H.H. (1996). A new learning algorithm for blind signal separation. Adv. Neural Inf. Proc. Syst..

[51] Oja E. (1989). Neural networks, principal components, and subspaces. Int. J. Neural Syst..

[52] Bell A.J., Sejnowski T.J. (1995). An information-maximization approach to blind separation and blind deconvolution. Neural Comput..

[53] Bell A.J., Sejnowski T.J. (1997). The “independent components” of natural scenes are edge filters. Vis. Res..

[54] Hinton G.E., Salakhutdinov R.R. (2006). Reducing the dimensionality of data with neural networks. Science.

[55] LeCun Y., Bengio Y., Hinton G. (2015). Deep learning. Nature.

[56] Isomura T., Toyoizumi T. (2018). Error-gated Hebbian rule: A local learning rule for principal and independent component analysis. Sci. Rep..

[57] Hyvärinen A., Pajunen P. (1999). Nonlinear independent component analysis: Existence and uniqueness results. Neural Netw..

[58] Yang H.H., Amari S.I. (1997). Adaptive online learning algorithms for blind separation: Maximum entropy and minimum mutual information. Neural Comput..

[59] Latham P.E., Nirenberg S. (2005). Synergy, redundancy, and independence in population codes, revisited. J. Neurosci..

[60] Amari S.I., Nakahara H. (2006). Correlation and independence in the neural code. Neural Comput..

[61] Berkes P., Orbán G., Lengyel M., Fiser J. (2011). Spontaneous cortical activity reveals hallmarks of an optimal internal model of the environment. Science.

[62] Sengupta B., Stemmler M.B., Friston K.J. (2013). Information and efficiency in the nervous system—A synthesis. PLoS Comput. Biol..

[63] Frémaux N., Gerstner W. (2016). Neuromodulated Spike-Timing-Dependent Plasticity, and Theory of Three-Factor Learning Rules. Front. Neural Circuits.

[64] Isomura T., Toyoizumi T. (2016). A Local Learning Rule for Independent Component Analysis. Sci. Rep..

[65] Kuśmierz A., Isomura T., Toyoizumi T. (2017). Learning with three factors: Modulating Hebbian plasticity with errors. Curr. Opin. Neurobiol..

[66] Isomura T., Sakai K., Kotani K., Jimbo Y. (2016). Linking neuromodulated spike-timing dependent plasticity with the free-energy principle. Neural Comput..

[67] Markram H., Lübke J., Frotscher M., Sakmann B. (1997). Regulation of synaptic efficacy by coincidence of postsynaptic APs and EPSPs. Science.

[68] Bi G.Q., Poo M.M. (1998). Synaptic modifications in cultured hippocampal neurons: Dependence on spike timing, synaptic strength, and postsynaptic cell type. J. Neurosci..

[69] Karnani M., Pääkkönen K., Annila A. (2009). The physical character of information. Proc. R. Soc. A Math. Phys. Eng. Sci..

[70] Annila A. (2016). On the character of consciousness. Front. Syst. Neurosci..

